# Thromboembolic Complications in Covid-19: From Clinical Scenario to Laboratory Evidence

**DOI:** 10.3390/life11050395

**Published:** 2021-04-27

**Authors:** Alberto Palazzuoli, Michela Giustozzi, Gaetano Ruocco, Francesco Tramonte, Edoardo Gronda, Giancarlo Agnelli

**Affiliations:** 1 Cardiovascular Diseases Unit, Department of Medical Sciences, Le Scotte Hospital, University of Siena, Viale Bracci, 53100 Siena, Italy; gmruocco@virgilio.it (G.R.); molex829@gmail.com (F.T.); 2 Internal Vascular and Emergency Medicine and Stroke Unit, University of Perugia, 06121 Perugia, Italy; michela.giustozzi@unipg.it (M.G.); giancarlo.agnelli@unipg.it (G.A.); 3 Department of Medicine and Medical Specialties, IRCCS Foundation Ca’ Granda Hospital, 20126 Milan, Italy; edogronda@icloud.com

**Keywords:** coagulation, thrombosis, COVID-19 infection, antithrombotic therapy

## Abstract

SARS-Cov-2 infection, a pandemic disease since March 2020, is associated with a high percentage of cardiovascular complications mainly of a thromboembolic (TE) nature. Although clinical patterns have been described for the assessment of patients with increased risk, many TE complications occur in patients with apparently moderate risk. Notably, a recent statement from the European Society of Cardiology (ESC) atherosclerosis and vascular biology working group pointed out the key role of vascular endothelium for the recruitment of inflammatory and thrombotic pathways responsible for both disseminated intravascular coagulation and cardiovascular complications. Therefore, a better understanding of the pathophysiological process linking infection to increased TE risk is needed in order to understand the pathways of this dangerous liaison and possibly interrupt it with appropriate treatment. In this review, we describe the histological lesions and the related blood coagulation mechanisms involved in COVID-19, we define the laboratory parameters and clinical risk factors associated with TE events, and propose a prophylactic anticoagulation treatment in relation to the risk category. Finally, we highlight the concept that a solid risk assessment based on prospective multi-center data would be the challenge for a more precise risk stratification and more appropriate treatment.

## 1. Introduction

Coronavirus disease 2019 (SARS-Cov-2) is a new viral infection causing acute respiratory distress syndrome (ARDS), which was first detected in December 2019 in Wuhan, China. This infectious disease quickly spread to almost all countries, causing the most dangerous pandemic since the Spanish flu in 1918–1920 [1]. One of the worst features of COVID-19 is a severe coagulopathy named COVID-19 induced coagulopathy (CAC) with an increased risk of thromboembolic complications and an incidence of venous thromboembolism (VTE) of about 25%. Injury of vascular endothelial cells infected with SARS-Cov-2 could trigger neutrophil attraction and immunocomplex formation. The contemporary activation of immune, inflammatory and coagulation pathways is consistent with the concept of immunothrombosis [2]. These two processes are initially triggered by a diffuse endothelial dysfunction induced by SARS-Cov-2 through angiotensin-converting enzyme (ACE-2) receptors and the Transmembrane protease serine 2 (TMPRSS2) that are currently recognized as specific sites by which the virus enters into the vascular system. The rare autoptic examinations of patients with COVID-19 shows extensive areas of inflammatory infiltration associated with interstitial oedema, thrombotic lesions and changes in the alveolar structure, with distinctive vascular features consisting of severe endothelial injury, called endothelialitis, associated with the presence of intracellular virus and disrupted cell membranes. Histologic analysis of pulmonary vessels in patients with COVID-19 showed widespread thrombosis with microangiopathy, including hyaline membrane formation, macrophages and monocyte infiltration [3,4,5].

Although these findings were observed mainly in patients older than 65 years, with previous associated diseases, they could also be seen in some younger subjects who experienced pulmonary complications with extensive parenchymal involvement requiring invasive ventilation care. A recent position paper by the European Society of Cardiology (ESC) working group of atherosclerosis and vascular biology focused attention on the endothelial damage as the primary organ target for thrombotic complications [6]. This complex scenario encompasses an altered immune and inflammatory response, oxidative stress status, impaired structural endothelium properties, and platelet activation. The histological picture is also mediated by both a specific lymphocyte population leading to an exacerbated immunity feedback, and by a preliminary coagulation pattern that predisposes subjects to intravascular thrombosis. Increased thrombotic activity may occur in other vascular districts such as coronary and cerebral vessels leading to acute coronary syndrome and stroke events, respectively [7,8]. Although these well-known notions have been reported in recent literature, those patients at an increased risk for developing immune and thromboembolic (TE) complications remains to be clarified. Another point to clarify is whether the current clinical laboratory and diagnostic approaches applied in COVID-19 patients is able to promptly recognize and eventually prevent related clinical deterioration. A better understanding of mechanisms linking infection to embolic complications is a challenge for future research. Notably, an altered signal of coagulation cascade and the specific investigation of molecular and protein signal dysfunction could result in an optimized treatment.

## 2. Methods

This is a narrative review focused on thrombotic complications related to SARS-Cov-2 infection including data that came from clinical randomized trials, controlled studies, meta-analysis, and position papers published in this field. We excluded from our paper case reports and single center studies. Terms and definitions searched were “risk factors”, “thrombosis”, “coagulation”, “vascular complications” and “antithrombotic therapy” for published papers appearing on Pub Med during a period ranging from December 2020 up to March 2021.

## 3. Risk Factors Pattern Suggestive of Adverse Outcomes

Epidemiological studies showed that elderly patients with a high comorbidity burden are much more prone to developing adverse events after COVID-19 infection. A history of cardiovascular diseases (CVD), or simply the presence of risk factors such as diabetes and hypertension, are associated with an adverse outcome [9]. Accordingly, the need for intensive care unit (ICU) admission and invasive ventilation support is more common in subjects with pre-existing CVD that suffer a mortality 5 to 10 fold higher compared to patients without CV risk factors [10,11]. Subjects with CV disease and high comorbidity burden are more likely to develop cardiac complications mainly due to increased TE complications and ARDS [12,13]. These findings have been confirmed in 5700 patients hospitalized in the New York City area, showing that CV risks are independent of race and geographic area [14].

In a meta-analysis including 75,000 subjects, hypertension, CVD, diabetes mellitus, smoking, chronic obstructive pulmonary disease, malignancy, and chronic kidney disease were the most prevalent underlying diseases related to hospitalization [15]. Of note, another analysis evaluating cardiac biomarkers showed that patients with CVD experienced increased levels of Troponin (TnT), N-terminal pro-B-type natriuretic peptide (NT-proBNP) and C reactive protein. The mortality rate during hospitalization was linearly related to both a history of CVD and TnT levels: it was relatively favorable in patients with known CVD without elevation of TnT levels, but unacceptably high during hospitalization for those with either underlying CVD or elevated TnT (69%) [16]. Current data demonstrate that myocardial injury is significantly associated with an adverse outcome of COVID-19, and this trend is likely associated with the presence of CVD. The study provides additional insights that TnT levels are significantly associated with levels of C-reactive protein (CRP) and NT-proBNP, linking myocardial injury to the severity of inflammation. Inflammation and the related cytokine storm may involve both respiratory and cardiovascular systems; in the latter, it can result in myocarditis, cardiac arrhythmias, acute coronary syndrome, pulmonary embolism and disseminated coagulopathy. Although these complications may be associated with significant TnT elevation, its increase could be simply due to impaired ventilatory exchanges and reduced oxygen delivery at a myocardial level. The contemporary elevation of natriuretic peptides and TnT increases the risk of invasive ventilation, acute heart failure, elevated filling pressure and severe arrhythmic complications and ICU mortality [17,18]. Clinical manifestations and cardiovascular complications may comprise a wide scenario related to the district in which immunothrombosis occurs; although pulmonary complications are much more frequent, other systems may be involved by virus spread. The culprit organs are those with elevated blood perfusion in which a high concentration of virus can pivot. Of note, coronary and cardiac districts, the cerebrovascular system, and kidneys are the sites in which the virus can cause clinical complication by direct toxicity or by endothelial dysfunction located in different sites leading to thrombotic events and multi-organ failure [19,20].

## 4. Autoptic Findings and Diagnostic Evidence

Recent autoptic studies showed alveolar damage to macrophages and monocyte infiltration, capillary luminal and mural fibrin deposition, hyaline thrombi on microvessels and thrombogenic vasculopathy as well as catastrophic microvascular injury syndrome. A few cases also reported thrombotic evidence in the large branches of pulmonary circulation [21]. These features suggest that many complications occur as a result of a TE event beyond the acute respiratory insufficiency related to infection damage and consequent extensive interstitial pneumonia.

All lung specimens from COVID-19 patients had diffuse alveolar damage with necrosis of alveolar lining cells, pneumocyte type 2 hyperplasia, and linear intra-alveolar fibrin deposition.

**Figure 1:**
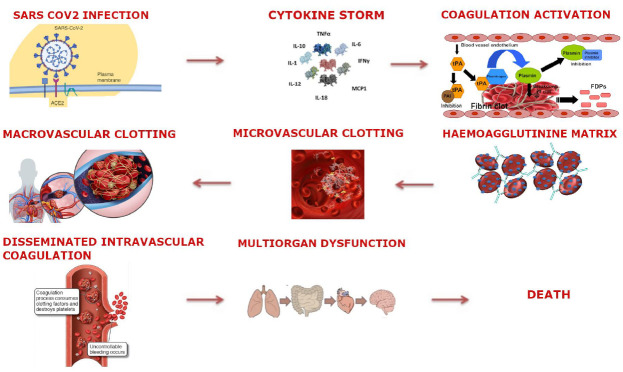
Summarizes the initial inflammatory activation leading to both coagulative cascade activation and vascular damage, two processes which lead to thrombotic hyperactivity with disseminated intravascular coagulation (DIC) in pulmonary and other districts. Because the infective process is primarily located in the lung, pulmonary circulation is prone to macroscopic thrombus formation and consequent pulmonary embolism (PE).

Diffuse pulmonary intravascular coagulopathy (PIC) induced by COVID-19 is a distinct process in respect to traditional disseminated intravascular coagulation (DIC). Indeed, it is recognizable by raised D-dimer, fibrinogen levels, and cardiac enzymes, respectfully reflecting pulmonary vascular bed thrombosis, fibrinolysis and emergent pulmonary hypertension induced ventricular stress. However, traditional DIC is comprised of platelet consumption with reduced levels and an altered coagulation cascade. A recent paper on 36 autoptic examinations describes a series of lesions including capillary congestion associated with interstitial oedema, necrosis of pneumocytes and diffuse inflammatory infiltration mainly composed of lymphocytes and macrophages. Platelet-fibrin thrombi in small arterial vessels were found in most of the cases examined and the current picture was associated with a relevant percentage of condensed fibrin [22]. This picture could configure an attempt of thrombus reparation with blunted vascular regeneration, and it highlights the importance of inflammation-platelet axis activation. Finally, a recent case series report comparing influenza pneumonia with COVID-19 related pneumonia demonstrated in both forms diffuse lung alveolar damage with perivascular T-cell infiltration. Interestingly, alveolar capillary microthrombi were nine times more prevalent in SARS-Cov-2 patients than in patients with influenza [23]. The histological examination also showed distinctive vascular features, consisting of severe endothelial injury associated with the presence of intracellular virus and disrupted cell membranes. Another distinctive feature of SARS-Cov-2 pneumonia is large pulmonary vessel involvement with extensive thrombosis into larger vessels (Figure 1).

Some reports have shown abnormalities within the pulmonary vasculature with contemporary presence of congestion and microthrombi. Moreover, electron microscopy studies reveal diffuse clots associated with endothelial injury and congestion with cell fragments and degenerate organelles in the lumen. Scanning electron microscopy of corrosion casts shows microvasculature of a larger diameter with an irregular surface, which may be due to endothelial injury and/or platelet aggregates or fibrin [22,23].

These autoptic findings appear to be confirmed by the clinical course of patients with a severe decrease of blood oxygen levels, submitted to invasive ventilation. On the basis of these topics, it is mandatory to investigate the thromboembolic risk in COVID-19 patients. Notably, neurological damage due to systemic hypoxia, microvascular thrombosis and vasoconstriction of cerebral vessels related to a septic status could deteriorate clinical conditions. Less common manifestations such as meningitis, encephalitis, cerebral bleeding, subarachnoid hemorrhage, and intracerebral hematomas may impair clinical scenarios and reduce consciousness and ideation [24].

## 5. Altered Molecular Signal and Coagulation Overexpression

Emerging evidence shows that severe SARS-Cov-2 infection can be complicated with abnormalities of blood coagulation and that these are related to a poor prognosis and an increased risk of intensive care unit admission [25]. The mechanisms underlying infection, inflammation and coagulopathy in SARS-Cov-2 disease have been poorly investigated, but likely reflect the course of similar viral infections, such as SARS and Ebola. In specific conditions characterized by altered inflammatory activity, together with immune response up-regulation, a pro-coagulation state prevails [22]. The pathophysiological mechanisms of SARS-Cov-2-associated coagulopathy appear to follow the Virchow’s Triad comprising of: (i) vascular endothelium damage, (ii) altered blood flow, and (iii) platelet function abnormalities [26]. Some laboratory tests are suggestive of increased thrombotic risk; lymphocytopenia associated with a mild increase of neutrophil counts and inflammatory markers may indicate an immune response dysfunction. Similarly, D-dimer and fibrinogen increase associated with plasminogen activator-1 inhibitor (PAI-1) activation are linked to a prothrombotic status. Reduced platelet counts, as the consequence of platelet activation and consumption, may be the trigger for thrombus formation in the microvascular district. An increased interleukine (IL-1, IL-6) level is the signal of systemic endothelial damage, and vasculitis occurrence in both arterial and venous systems [17,26].

In physiological conditions, the coagulation cascade activation recognizes three different pathways: the antithrombin system, the endothelial protein C system, and tissue factor (TF) pathway inhibitor. In acute sepsis, all of these pathways are inhibited because of impaired synthesis, on-going consumption and proteolytic degradation. Overexpression of tissue factors, activation of the C protein system, and the inhibition of physiological fibrinolysis processes represent the key mechanisms involved in this coagulopathy. Indeed, viral infection induces an overexpression of immune mediators such as cytokines, vascular and endothelial grow factors. The cytokine storm activates adhesion protein expression, platelet aggregation, C protein activation and fibrin formation [27,28]. The endothelial disruption results in a massive release of Von Willebrand factor and loss of the fibrinolytic function of these cells, facilitating the thrombus formation.

Therefore, the viral infection induces, per se, an uncontrolled generalized immune response, including the activation of many immune cells like T-lymphocytes, macrophages and dendritic cells responsible for a massive local release of pro-inflammatory cytokines and aggravation of endothelial injury [29]. The two-way association between systemic inflammation and activation of coagulation is named immunothrombosis [28,29]. The main mediators of immunothrombosis in patients with SARS-CoV-2 include interleukin-6 (IL-6), interleukin-1β (IL-1β) and tissue factor (TF) [19]. IL-6 can induce TF expression in mononuclear cells and initiates coagulation activation and thrombin generation. TNF-α and IL-1β are the main mediators driving a depression of endogenous anticoagulant pathways. Neutrophils, in turn, stabilize microthrombi through the release of neutrophil extracellular traps (NETs) and neutrophil elastase that immobilize inflammatory cells and promote intravascular fibrin formation [30].

Altered blood flow in patients with COVID-19 disease is linked to hyper-viscosity that predisposes subjects to thrombosis and induces endothelial damage and dysfunction [31]. Intravascular fibrin formation is the major determinant of blood viscosity. In the microcirculation, this turbulent blood flow results in microthrombosis that acts as a perpetrator of abnormal blood flow and facilitates additional thrombosis [32,33].

The loss of platelet function increases the risk of a prothrombotic state. Platelet activation is induced by both the viral infection and indirectly by NETs [34]. A further coagulation derangement could occur because ACE2 is involved in the activation of the tissue plasminogen activator (tPA) and angiotensin-II has an important role in the release of the PAI-1 from endothelial and smooth muscle cells [35]. Indeed, the loss of ACE2 alters the PAI-1/tPA balance, favoring a prothrombotic state and platelet activation. Indirectly, NETs lead to platelet activation via toll-like receptors on platelets and other cells, linking inflammation, coagulation and thrombosis in multiple conditions [36]. ACE2 has local anti-inflammatory functions by inactivating kallikreins and bradykinins. A loss of ACE2 function can lead to an overactivation of the kallikrein-bradykinin pathway, leading to inflammation and oedema [36,37]. Since the kallikrein-bradykinin system is closely linked to the coagulation system via the activation of factor XII, the system activation may drive towards potential intravascular coagulation. This process may be amplified further by hemagglutinin esterase contained in S-protein spikes. The enzyme acts on sialic acid (SA) glycoconjugate receptors, mediating the critical preliminary step of viral attachment prior to fusion with an ACE2 receptor and replication in a host cell. The binding of the SARS-Cov-2 virus to SA and CD147 receptors, abundant on red blood cells and endothelial cells, is well established, and it leads to a diffuse microvascular obstruction [37,38].

**Figure 2:**
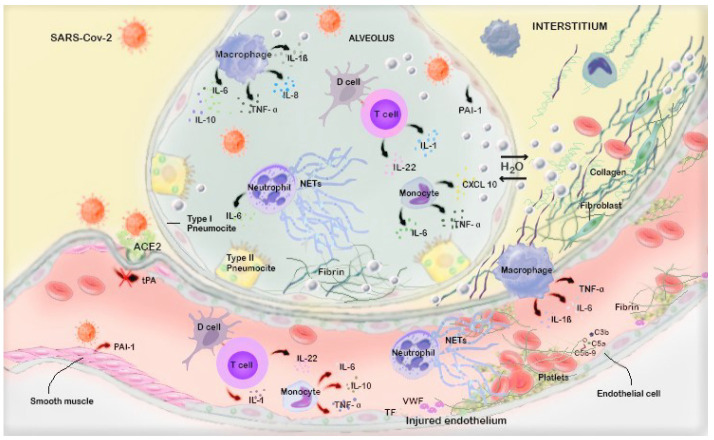
Describes the potential pathophysiological features related to SARS-Cov 2 infection. Inside the vascular district, Covid-19 could penetrate through ACE receptors, causing an immune response with leukocyte and neutrophil attraction, cytokine storm and complement activation. Current processes induce platelet activity, endothelial dysfunction pericytes migration and Von Willebrand factor overexpression. Loss of endogenous fibrinolytic capacity, due to C-protein activation, tissue plasminogen factor (TPA) inhibition and tissue plasminogen activator (PAI-1) are the primary drivers for thrombus formation. At an alveolar level, the immune inflammatory response amplified by cytokines, causes pneumocyte barrier alteration, increased permeability and water insertion. Finally, at interstitial level, inflammatory overdrive increases vascular and collagen growth factor expression with fibroblasts retrievement, interstitial oedema and fibrosis onset.

Finally, a dysregulated complement activation has been demonstrated to be associated with thrombogenesis by promoting platelet activation in endothelial cells and increasing tissue factor and Von Willebrand expression [38,39] (Figure 2).

## 6. Could Laboratory Assays Be Useful for Thromboembolic Risk Stratification?

Many patients with severe COVID-19 presentation experience some typical laboratory findings indicating hypercoagulability. Coagulopathy in patients with COVID-19 is associated with an increased risk of death and in-hospital thromboembolic events [40,41]. Although the initial prothrombotic cascade in patients with COVID-19 is similar to other types of sepsis, in patients with COVID-19 the relationship between increased thrombogenicity and infection seems to have some different features. Specifically, patients with significantly increased inflammatory biomarkers (CRP) and erythrocyte sedimentation velocity (VES) are much more prone to developing consumptive coagulopathy and diffuse intravascular coagulation (DIC). The most common pattern of coagulopathy observed in these patients is primarily characterized by increased D-dimer and fibrin/fibrinogen degradation product, a mild prolongation of activated partial thromboplastin time (aPTT) and/or prothrombin time (PT), and a relatively modest decrease in platelet count [42,43]. Accordingly, most COVID-19 patients would not be classified as having DIC according to the International Society on Thrombosis and Haemostasias’ (ISTH) definition. Moreover, recent evidence suggests that COVID-19 infection infrequently leads to bleeding diathesis, despite abnormal coagulation parameters [44]. In a recent meta-analysis including 60 studies, significantly higher PT, D-dimer and fibrinogen levels and a lower platelet count were observed in 5487 patients with severe COVID-19 compared to 9670 patients with mild COVID-19 [45]. Additionally, patients who survived had higher PT and D-dimer levels, and a lower platelet count, compared to patients who did not survive. Conversely, an elevated D-dimer level correlated to an elevated C-reactive protein at initial presentation, which was predictive of pulmonary embolism and DIC in COVID-19 and closely related to an inflammatory pattern in patients admitted in ICU [46]. Additionally, a high level of fibrinogen and D-dimer indicates a progressive severity of COVID-19 infection leading to coagulation activation, cytokine storm and multiorgan failure [47,48]. A D-dimer value more than 6 times above the normal limit is a strong predictor of thrombotic events and poor prognosis in these patients [49].

In terms of laboratory workup, monitoring the D-dimer, PTT, platelet count, and fibrinogen levels for early identification of severe COVID-19 patients as well as consideration of more aggressive treatment is recommended [50]. The ISTH recommendation suggests using laboratory parameters, D-dimer, PTT, platelet count and fibrinogen at admission, to stratify patients with COVID-19 disease who require hospitalization [44,50]. Specifically, the patient needs to be hospitalized in cases with D-dimer levels > 3–4 times above normal, prothrombin time prolonged, platelet count < 10,000 and fibrinogen < 2.0 g/L. However, further studies are needed to validate this algorithm in clinical practice.

In patients with COVID-19, other laboratory parameters that might be important for coagulopathy are increased lactate dehydrogenase and ferritin concentration, without signals of haemolysis or presence of schistocytes [20,45]. All these laboratory changes suggest that COVID-19 coagulopathy may be an association between low-grade DIC and localized pulmonary thrombotic microangiopathy. Furthermore, while antithrombin is moderately lower in these patients, factor VIII, IL-6, CRP, Von Willebrand, and plasma viscosity are higher, suggesting a strong association between systemic inflammatory response and thrombosis [43,51].

A recent trial demonstrates the specific role of the combined endothelial and platelet activation; significant elevation of P selectin Von Willebrand factor (VWF) and soluble thrombomodulin were associated with both in-hospital mortality and prolonged hospitalization [49] (Table 1). Current reports demonstrate a contemporary platelet activation associated with an endothelial dysfunction and related thrombotic propensity.

Accordingly, in patients with COVID-19 disease, viscoelastic properties evaluated by specific tools such as thromboelastography (TEG) and rotational thromboelastometry (ROTEM) revealed increased clot-strength (CS), demonstrated by elevated platelet aggregation, elevated D-dimer levels, and hyperfibrinogenemia [52,53]. Current analysis is usually performed in patients with coagulation defects and polymorphism variance; no prospective data are extensively available in COVID-19 infection, therefore caution is needed regarding the interpretation and application of these assays in this setting. Present data suggest that a combined network involving inflammation as the primary trigger for coagulation and platelet activation as well as endothelial dysfunction and damage are the main features for thrombotic complications.

## 7. Which Antithrombotic Treatment Is the Most Appropriate?

Given the evidence that patients with COVID-19 disease are at increased risk of venous thromboembolism, several consensus guidelines and consensus documents are available to guide clinicians in the decision-making process for these patients [27,50,54]. However, the practical Guidelines are primarily based on practical experience and epidemiological data rather than on interventional randomized trial and prospective study design [25,50]. Up until now, the identification of COVID-19 patients at risk for clinical deterioration and thrombotic complications has been based on patients’ history and previous algorithms built before the pandemic era [51,55,56].

In all patients with COVID-19, in the absence of contraindications, the use of thromboprophylaxis is recommended. Low-molecular weight-heparin (LMWH) (e.g., enoxaparin 40 mg once daily or dalteparin 5000 UI daily) at standard dose is the recommended amount for venous thromboembolism prevention in these patients. Unfractionated heparin should be preferred in patients with severe renal failure (creatinine clearance less than 30 mL/min) or in patients at high risk of bleeding, or those undergoing an emergency procedure. Limited evidence is currently available for the use of fondaparinux. These recommendations are based on several studies that have shown a high incidence of TE complications, both pulmonary embolism and deep vein thrombosis, in hospitalized patients with COVID-19 disease. In fact, the incidence of venous thromboembolism in these patients seems to range from 24% to 27%, which means that about one third of patients with COVID-19 will have an episode of venous thromboembolism during the disease [27,57]. The incidence of pulmonary embolism is about 16.5%, while that of deep vein thrombosis varies from 7% to 14%. This incidence increases with the severity of the disease, and it is higher in patients admitted to intensive care units. Accordingly, some scientific societies support the use of intermediate LMWH (e.g., enoxaparin 40 mg twice daily or enoxaparin 1 mg/kg/day) in selected severe and critically ill COVID-19 patients [18,25]. Extended thromboprophylaxis with LMWH up to 45 days post-hospital discharge should be considered in hospitalized COVID-19 patients at high risk for venous thromboembolism defined as D-dimer levels less than two times above the reference range, advanced age, long stay in the intensive care unit, prior VTE episode, cancer history, prolonged immobility, thrombophilia, and VTE score ≥ four [58,59] (Table 2).

Since no randomized trial on the management of VTE in patients with COVID-19 disease has been published so far, these recommendations are mainly based on expert opinions and observational studies. However, the observational studies published differ in terms of use of LMWH or unfractionated heparin and dosage, ranging from prophylactic to intermediate or therapeutic dose. Indeed, most data came from epidemiological data obtained from observational studies, and few results can be achieved from randomized controlled trials [44,60]. Therefore, caution is necessary when applying these results in clinical practice. The main question of whether the traditional scores purposed for common thromboprophylaxis are applicable, accurate enough and reproducible in the COVID-19 setting, is mandatory in order to categorize the risk of these patients [61,62].

In hospitalized COVID-19 patients with confirmed venous thromboembolism, the aforementioned consensus guidelines or recommendations advocate the use of full therapeutic doses of anticoagulant drugs [60,63]. LWMH is the most preferred agent, followed by unfractionated heparin in all patients without contraindications.

However, currently there is no clear evidence about the optimal dose regimen. LMWH was suggested as the first option instead of unfractionated heparin (UFH), as UFH requires more frequent injections and increases the risk of transmission of SARS-Cov-2. Nevertheless, an increased dose is probably preferred in those patients with thromboprophylaxis and more severe COVID-19 presentation [58].

Unfortunately, current data do not support the use of thromboprophylaxis, guided by common biomarkers such as D-dimer and fibrinogen; however, observational trials suggest that the early use of anticoagulant for hospitalized patients is associated with reduced adverse events. In a recent study of 1240 patients with COVID-19, the use of a prophylactic dose introduced during hospitalization, or anticoagulant therapy with a therapeutic dose administered prior to hospitalization, reduced the onset of pulmonary embolism, suggesting the importance of starting preventive treatment as soon as possible [59]. Of note, all pulmonary embolism diagnoses in this study were confirmed by computed tomography pulmonary angiography. In addition, thromboprophylaxis and therapeutic anticoagulation should be withheld in the case of active bleeding or severe thrombocytopenia, and if abnormal PT or PTT is a contraindication to thromboprophylaxis. When pharmacological thromboprophylaxis is contraindicated, mechanical thromboprophylaxis should be considered.

A rationale for all of the above recommendations is also partly based on the benefits of anticoagulant therapy reported in terms of improved survival in these patients. Of note, in a recent study of 2773 hospitalized patients, a significant increased probability of in-hospital survival was observed in patients treated with anticoagulation, especially among patients that required mechanical ventilation [60]. A possible explanation of the beneficial effect of heparin and its derivatives in patients with COVID-19 is based on the additional anti-inflammatory action. Since the severe inflammatory response in COVID-19 is the main trigger of coagulopathy, the concept of using heparin and its derivatives seems clinically validated as reducing thrombotic and inflammatory pathways can improve the outcome in these patients [61].

Finally, the role of direct oral anticoagulants (DOACs) in COVID-19 patients is currently unknown. In a study of 1039 hospitalized COVID-19 patients, 32 were on treatment with direct oral anticoagulants. DOACs plasma levels were measured in patients concomitantly treated with DOACs and antiviral agents (lopinavir, ritonavir, or darunavir). A significant increase of DOACs plasma levels was observed in these patients, suggesting replacement with alternative parenteral antithrombotic strategies to reduce the risk of bleeding. Accordingly, in 3625 hospitalized patients, in a head to head comparison between DOACs and heparin, an adjusted logistic regression analysis demonstrated a significant decrease in mortality with prophylactic use of both apixaban (odds ratio 0.46) and enoxaparin (OR = 0.49) [64,65]. However direct oral anticoagulants (DOACs), such as apixaban, have numerous potential benefits, although their use in intensive care units (ICU) needs further investigations. Furthermore, despite the lack of support for DOACs in the most recent CHEST 25 guidelines for the prevention and treatment of COVID-19-induced coagulopathy, any increase of adverse events rate was observed with the use of apixaban including major bleeding [65].

Only one anticoagulant class is not recommended for thromboprophylaxis, which is direct vitamin K antagonist (VKA). Two large studies have demonstrated an increased risk for bleeding associated with current treatment in both hospitalized and non-hospitalized patients. The increased adverse event risk appears much more noticeable for frail and elderly patients [66,67].

## 8. Limitations

Despite current papers describing the usual clinical manifestations related to COVID-19 infection focusing on thromboembolic complications, the underlying mechanisms are not completely understood. The concept of immunothrombosis is partially elucidated, and ongoing studies are searching for the complex mechanisms linking inflammatory and immune response with altered thrombotic processes. Indeed, the disease’s underlying cellular and molecular mechanisms are still poorly understood. Therefore, laboratory analysis does not always recognize the group at increased risk, and new biomarkers strictly related to coagulation cascade may probably increase the prediction accuracy. However, the aim of this review is to report a much more available blood test capable of defining thrombotic risk in clinical practice. The last SARS-Cov2 virus variants may have different presentation, and thromboembolic risk could change in relation to host diffusion and virus affinity with cardiovascular receptors. Finally, the treatment described is based on the clinical trials published until now, although several ongoing studies are testing other anticoagulant drugs with various doses in different clinical setting. Anticoagulation treatment could vary in relation to infection severity, preliminary patients’ conditions, and hospitalized vs. home treated patients.

## 9. Conclusions

TE complications are intrinsic features of SARS-Cov-2 syndrome, and are responsible for a significant percentage of clinical deteriorations. The key components are represented by altered immune response, cytokine storm and endothelial dysfunction inducing an altered coagulation process, vasoconstriction and reduced fibrinolytic activity. Serial measurement of laboratory assays together with clinical screening may help in recognizing patients with an elevated risk in order to begin an appropriate anticoagulant therapy. An accurate score accounting for detailed laboratory patterns and clinical evaluation will be a challenge for future research, to enable the reduction of COVID-19 related events.

## Figures and Tables

**Table 1:** Laboratory findings in patients with COVID-19 disease: the typical laboratory pattern reflecting infection.

Laboratory Variable	Comments	
D-dimer	🡹🡹🡹	Markedly elevated
FDPs	🡹🡹	Elevated
Fibrinogen	🡹🡹	Elevated
aPTT	🡸🡺 (🡹)	In normal range OR slightly prolonged
PT	🡸🡺 (🡹)	In normal range OR slightly prolonged
Platelet count	🡸🡺 (🡻) (🡹)	Normal OR mildly decreased in most cases
Von Willebrand Factor	🡹🡹🡹	Markedly elevated
P-selectin	🡹🡹	Elevated in very severe COVID-19 group
Thrombomodulin	🡹(🡹🡹)	Slightly elevated; Elevated in severe COVID-19 manifestations
Lactate dehydrogenase	🡹🡹	Elevated
C-reactive Protein	🡹🡹	Elevated in diffuse COVID-19 pneumonia
Ferritin	🡹🡹	Elevated in severe COVID-19 manifestations

FDPs: fibrinogen degradation products; aPTT: thrombolastin time; PT: prothrombin time.

**Table 2:** Potential advantages and pitfalls of different anticoagulant therapies and regimens in patients with COVID-19.

	Advantages	Disadvantages	Comments
UFH	- Short half-life - Antidote available	- Need of laboratory monitoring - Intravenous administration	Consider in patients with acute kidney injury or creatinine clearance <15 mL/min, at high risk of bleeding or needing imminent procedures.
LMWH	- Short half-life - Regimens may be modified according to body weight, severe thrombocytopenia, or worsening renal function - Less heparin-induced thrombocytopenia compared UFH - Less drug–drugs interactions compared to oral anticoagulants	- Lack of antidote	In-hospital agent of choice, administered as prophylaxis treatment or after TE event at home
APIXABAN	- Fixed doses - Lack of monitoring	- Potential drug–drug interaction - Lack of reversal at some centers - Longer half-lives - Cost implications	Ideal for outpatient management

## Data Availability

Not applicable.
